# Resveratrol and Angiogenin-2 Combined With PEGDA/TCS Hydrogel for the Targeted Therapy of Hypoxic Bone Defects via Activation of the Autophagy Pathway

**DOI:** 10.3389/fphar.2021.618724

**Published:** 2021-04-13

**Authors:** Dehui Fan, Hengping Liu, Zhenning Zhang, Meiyi Su, Zhixian Yuan, Ying Lin, Shuquan Yang, Wenqiang Li, Xintao Zhang

**Affiliations:** ^1^ The Fifth Clinical College of Guangzhou University of Chinese Medicine Guangzhou, Guangdong Second Traditional Chinese Medicine Hospital, Guangzhou, China; ^2^ Beijing University of Chinese Medicine Third Affiliated Hospital, Beijing, China; ^3^ Engineering Technology Research Center for Sports Assistive Devices of Guangdong, Guangzhou Sport University, Guangzhou, China; ^4^ Department of Sports Medicine and Rehabilitation, National and Local Joint Engineering, Research Center of Orthopaedic Biomaterials, Peking University Shenzhen Hospital, Shenzhen, China

**Keywords:** resveratrol, ANG2, autophagy, hypoxia condition, vascularization, bone defect

## Abstract

The guarantee of cell survival under hypoxic conditions and rapid vascularization is a key in tissue engineering strategies for treating bone defects. Our study aimed to establish the protective role of bone marrow mesenchymal stem cells (BMSCs) and human umbilical vein endothelial cells (HUVECs) in hypoxic conditions and realize rapid vascularization in bone defects. Resveratrol (Res), a non-flavonoid polyphenolic compound, and angiopoietin-2 (ANG2), a vascular activating factor, were applied to enhance BMSC and HUVEC survival, osteogenesis, and angiogenesis. The morphology, autophagy, viability, apoptosis, cycle, and osteogenic differentiation of BMSCs treated with Res were analyzed. The results indicated that Res could improve BMSC survival and differentiation via the autophagy pathway under hypoxic conditions. In addition, Res maintained HUVEC growth and proliferation in a hypoxic and ANG2 double-adverse environment via the autophagy pathway. To simulate a relatively hypoxic environment, small-aperture PEGDA/TCS hydrogels containing Res and ANG2 were prepared. BMSCs were cultured in the PEGDA/TCS scaffold and transplanted into a large tibial defect. CD31 immunofluorescence showed that the density and size of new blood vessels in the bone defect were significantly enhanced by ANG2 and Res at 8 weeks after surgery. H&E, Masson, and immunohistochemical staining results indicated that ANG2 combined with Res could promote new bone formation in defects. All these results suggested that Res combined with ANG2 may be a novel strategy for the targeted therapy of hypoxic bone defects with tissue engineering scaffolds.

## Introduction

Bone defect reconstruction is a continuous and complex biological process involving many functional cells, the extracellular matrix and various factors (Drosse et al., 2008; Horner et al., 2010). Tissue engineering technology provides new strategies for bone defect repair and regeneration (Cheng et al., 2019; Zhou et al., 2020). Tissue engineering scaffolds provide locations for cell growth and proliferation and bone marrow mesenchymal stem cell (BMSC) differentiation for new bone formation (Qasim et al., 2019; Liu et al., 2020). Among multitudinous tissue engineering scaffolds, hydrogel scaffolds have been widely investigated in bone tissue engineering (Luckanagul et al., 2016; Zhang et al., 2016; Sivashanmugam et al., 2017; Kim and Lee, 2020). Hydrogels are generally composed of hydrophilic polymers, which could mimic the bone tissue extracellular matrix, thus presenting a powerful ability to support cells growth and osteogenesis. However, hydrogels served as bone tissue engineering often fails to achieve bone defect repair due to the severely hypoxic microenvironment. During this hypoxic period, belated vascularization and BMSC or endothelial cell ischemic necrosis contribute to the failure of bone defect repair (Malda et al., 2004; Sathy et al., 2019; Wolff et al., 2019). To overcome the challenge, many researches focus on how to achieve vascularization rapidly, such as addition angiogenic factor and active ion to active angiogenesis pathway (Wu et al., 2012; Quinlan et al., 2015). However, the research did not consider too much how to keep the cells alive before angiogenesis. Here, a successful bone defect repair scaffold should depends on two aspects: whether tissue-engineered scaffolds can both achieve rapid vascularization and maintain functional cell survival until vascularization (Peng et al., 2019).

Cell (tissue) autophagy is a highly conserved, selective degradation process in cells that can eliminate abnormally accumulated long-lived proteins and damaged organelles (Denton and Kumar, 2019). Autophagy can prevent damaged cells and tissues from accumulating in the body and decrease metabolic stress and toxicity to increase the survival of cells and organs in hypoxic environments, which is important for maintaining cell stability, renewing cellular components, and maintaining a normal physiological status (Mazure and Pouysségur, 2010).

Angiopoietin-2 (ANG2) belongs to the ANG family and plays an important role in regulating vascular endothelial remodeling via the Tie2 signaling pathway (Thomas and Augustin, 2009). It plays a crucial role in the early stage of endothelial micro-vascular formation. The ANG2–Tie2 signaling pathway can enhance vascular permeability, increase connexin disassociation, activate endothelial cells, and create a pro-inflammatory environment (Veikkola and Alitalo, 2002; Scharpfenecker et al., 2005; Hakanpaa et al., 2015; Yang et al., 2016). Resveratrol (Res), a naturally occurring phytoalexin produced by some spermatophytes, possesses anti-inflammatory and anticancer properties (Frémont, 2000; Bhat et al., 2001). Thus, Res is required for exogenous ANG2 to increase the survival of endothelial cells before vascularization.

**SCHEME 1:**
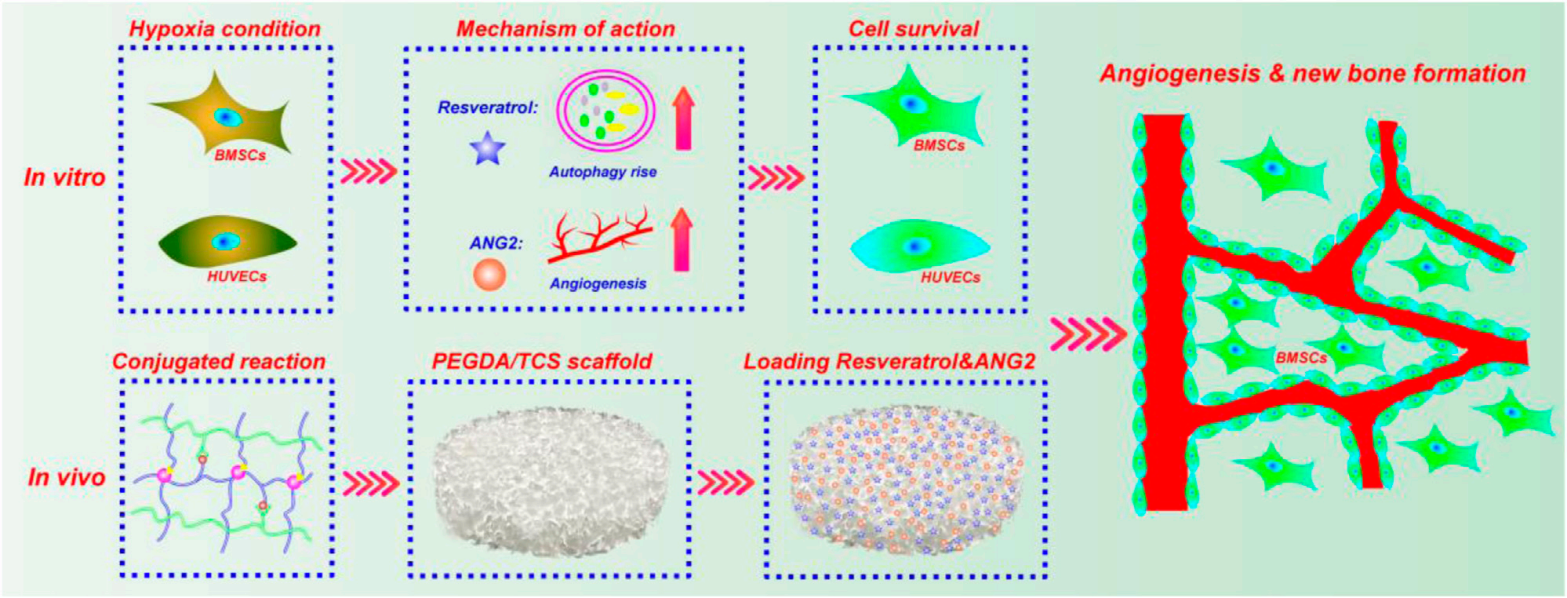
Schematic illustration of the synergistic effect of Res and ANG2 in the targeted treatment of bone defects under hypoxic conditions.

In this study, we established a hypoxic cellular environment to simulate the survival, osteogenic differentiation, and vascularization of BMSCs and human umbilical vein endothelial cells (HUVECs). We investigated the ability of Res to increase BMSC autophagy in a hypoxic environment to further regulate the physiological functions of BMSCs, including proliferation, survival, apoptosis, and differentiation. The regulation of endothelial cell proliferation and survival in a hypoxic environment by Res combined with ANG2 through the autophagy pathway was also explored. Finally, hydrogels with small apertures and excellent biocompatibility were prepared to simulate relatively hypoxic PEGDA/TCS tissue engineering scaffolds. After they were loaded with Res and ANG2 and co-cultured with BMSCs *in vitro*, they were implanted into a large-segment tibial bone defect in rats to investigate the ability of Res and ANG2 in repairing large-segment bone defects by using histology, immunohistochemistry, and immunofluorescence. As displayed in **Scheme 1**, this study will provide a new strategy for applying combinations of autophagy agonists and angiogenic drugs to treat large-segment bone defects and promote angiogenesis using tissue-engineered bone repair scaffolds.

## Experimental Section

### Synthesis of PEGDA/TCS Hydrogel

Poly (ethylene glycol) (PEG, Mn = 6,000, Sijia Biological Technology Co., China) was modified into PEGDA according to our previous study (Hu et al., 2018). Chitosan (CS, 200–800 cps, degree of deacetylation ≥85%), 2-hydroxy-4’-(2-hydroxyethoxy)-2-methylpropiophenone (Irgacure 2,959) and sodium beta-glycerophosphate were purchased from Sigma-Aldrich. Thiolated chitosan (TCS) was synthesized by our previous method (Li et al., 2017). First, PEGDA (1.5 g) was dissolved in 5 ml of deionized water, and TCS (0.05, 0.1, 0.2, 0.4 g) was dissolved in 5 ml of 0.8 wt% acetic acid solution separately and independently. The pH value was adjusted to approximately neutral by 50 wt% sodium *β*-glycerophosphate aqueous solution. Then, the two solutions were mixed according to the required volume with magnetic stirring, and then 0.1% (w/v) Irgacure 2,959 as a photoinitiator was added to the mixed solution to form a homogeneous solution with the designed final concentration (15 wt% PEGDA and 0.5, 1.0, 2.0, 4.0 wt% TCS). Second, the obtained solution was injected into the mold and exposed to UV irradiation (365 nm, 50 mW/cm^2^) for 5 min to form a PEGDA/TCS composite hydrogel. Finally, the resulting composite hydrogel was immersed in 3% (m/v) Na_2_SO_4_ solution for 30 min and converted to PEGDA/TCS DN (double network) hydrogel. The obtained hydrogels were washed three times with distilled water to remove the reaction residue and salts on the surface of the hydrogel for purification (Huang et al., 2019).

### PEGDA/TCS Hydrogel Characterization

Mechanical testing: Compression tests were carried out on a dynamic universal testing machine at 25°C. The samples were prepared as cylinders with a diameter of 10 mm and a height of 8 mm in wet condition, and the compression rate was set to 5 mm/min. In addition, the cycling compression test was conducted between a strain ranging from 0 to 60% at a rate of 2 mm/min for 20 cycles.

Fourier-transform infrared spectroscopy (FTIR): The dry PEGDA/TCS hydrogel was analyzed using a Bruker FTIR system from 4,000 to 400 cm^−1^.

Morphological examination: Freeze-dried PEGDA/TCS hydrogels were cryogenically fractured in liquid nitrogen and sputter-coated with gold before observation. Then, the morphology of the hydrogel was observed by scanning electron microscopy (SEM).

### BMSC Isolation, Cultivation and Identification

BMSCs were isolated from the femur and tibia of Sprague-Dawley (SD) rats (Liu et al., 2013) (4 weeks old, weighing 50–65 g). After the epiphysis was removed, the bone marrow tissue was flushed out from the femur and tibia by complete DMEM. To collect a homogeneous cell suspension, the marrow was repeatedly pipetted. Then, the cell suspension was seeded in a 25 cm^2^ cell culture flask and cultured in a humid atmosphere at 37°C with 5% CO_2_. The medium was renewed every two days, and the remaining adherent cells (defined as BMSCs) were digested by 0.25% trypsin EDTA solution upon reaching 90% confluence. Passage-three cells were used in subsequent experiments. For identification, BMSCs were digested with 0.25% trypsin and washed using PBS containing bovine serum albumin (BSA, 20%). The cells were subsequently re-suspended in complete medium in a single-cell suspension at a density of 5×10^6^ cells/mL. Eppendorf (EP) tubes were used to contain the single-cell suspension, and each tube was filled with 200 µL of the suspension (5×10^6^ cells/mL). For flow cytometric detection, monoclonal antibodies against CD29 (FITC-conjugated, ThermoFisher, AB_2,572,449), CD34 (FITC-conjugated, ThermoFisher, AB_11,154,336), CD44 (FITC-conjugated, ThermoFisher, AB_2,538,912), and CD45 (FITC-conjugated, ThermoFisher, AB_2,572,455) and fluorescein isothiocyanate-labeled mouse IgG (all BD Biosciences, Franklin Lakes, NJ, United States) were added to the EP tubes, and incubated at 4°C for 60 min. Then, the cells were washed with PBS containing 3% fetal bovine serum (FBS) 3 times and re-suspended following centrifugation at 120 g at 4°C for 5 min and subsequently fixed with 200 µL of polyoxymethylene (4%) at 4°C for 1 h. A flow cytometer and Cell Quest software (version 5.1; BD Biosciences) were used to analyze the cell phenotype. Before the experiment, BMSCs were preserved in liquid nitrogen, and when needed, BMSCs were revived as per the normal protocol. For the anoxic environment, BMSCs were washed with PBS under sterile conditions and then incubated for 20 h under hypoxic conditions (1% O_2_). Hypoxia was produced using a modular incubator gas chamber (mic-101, Billups-Rothenberg Co.). After allowing the culture media to degas for 1 h, the chamber was re-flushed in the same manner. All reagents used for cells treated under hypoxic conditions were also flushed with N_2_ before use.

### GFP-LC3 Plasmid Transfection and Fluorescence Staining

For the transfection experiments, GFP-LC3 was transfected into BMSCs using Lipofectamine 2000 (Invitrogen) according to the supplier’s protocol. The plasmid was purchased from Nanjing Sciben Biotech Co., Ltd. After the experiment, the cells were fixed in 4% PFA for 45 min. The LC3 puncta were observed by fluorescence microscopy (BX51, Olympus).

### Reactive Oxygen Species Level Analysis

ROS were detected using a dihydroethidium (DHE) fluorescence assay. BMSCs (1×10^5^ cells/mL) were seeded in a 2-cm Petri dish and cultured for 12 h. Cells were then treated for 24 h with 3-methyladenine (3-MA), Res and Res/3-MA at final concentrations of 5 mM, 50 μg/ml, and 50 μg/ml/5 mM, respectively. Then, the BMSCs were incubated with DHE for half an hour, and fluorescence microscopy (BX51, Olympus) was utilized to visualize changes in fluorescence intensity.

### BMSC and HUVEC Survival and Proliferation

MTT assay: BMSCs (1×10^4^ cells per well) were seeded in 96-well tissue culture plates for 24 h and then incubated for 24 h with 3-MA, Res and Res/3-MA at final concentrations of 5 mM, 50 μg/ml, and 50 μg/ml/5 mM. After incubation for 24 h, 20 μL/well MTT solution (5 mg ml^−1^ in PBS) was added to each well. After 4 h, the culture medium was removed and replaced with 150 μL/well DMSO. The absorbance of the DMSO solution at 570 nm was measured by a microplate reader.

Real-time growth curve: Cellular behavior was further investigated by a real-time cell analysis (RTCA) system (iCELLigence, ACEA Biosciences, Inc.). BMSCs were seeded in cell cultures with gold microelectrodes. The time point at which the cells attached and the medium was replaced with fresh medium was described as 0 h in this study. The cells were incubated for 20 h with 3-MA, Res and Res/3-MA at final concentrations of 5 mM, 50 μg/ml, and 50 μg/ml/5 mM, respectively. The cell growth curve was recorded automatically by RTCA. Similarly, HUVECs were incubated for 20 h with ANG2, Res/ANG2 and Res/ANG2/3-MA at final concentrations of 100 ng/ml, 50 μg/ml/100 ng/ml, and 50 μg/ml/100 ng/ml/5 mM, respectively.

### Acridine Orange-Ethidium Bromide Live/Dead Staining

To visualize the viability of BMSCs and HUVECs treated with different drugs, BMSCs, and HUVECs (1×10^4^ cells/well) were seeded in 96-well tissue culture plates for 24 h. After treatment with Res, 3-MA, and ANG2 (50 μg/ml, 5 mM, 100 ng/ml) for 24 h, cells were stained with 10 μL of a mixture of AO (100 μg ml^−1^), and EB (100 μg ml^−1^), and the cells were immediately examined under a fluorescence microscope (BX51, Olympus).

### Cell Apoptosis Assay and Cell Cycle

BMSCs were treated with 3-MA, Res, and Res/3-MA at final concentrations of 5 mM, 50 μg/ml, and 50 μg/ml/5 mM for 10 h in a six-well plate. After they were washed and centrifuged, the harvested cells were stained with an Annexin V-FITC/propidium iodide (PI) Apoptosis Detection Kit for 15 min at room temperature and then analyzed by a flow cytometer. In each experiment, 10,000 events per sample were recorded.

The effects of drugs on the cell cycle distribution were further examined by flow cytometry. BMSCs were separately treated with 3-MA, Res, and Res/3-MA at final concentrations of 5 mM, 50 μg/ml, and 50 μg/ml/5 mM for 24 h. Subsequently, cells were collected and fixed with 75% ethanol overnight at −20°C. The fixed cells were then stained with PI for approximately 1 h in darkness. Finally, the stained cells were measured with a flow cytometer, and 10,000 events per sample were recorded for each experiment.

### Alkaline Phosphatase and Alizarin Red S Staining

ARS and ALP staining were used to evaluate the extent of mineral deposition by BMSCs. The samples seeded with BMSCs which is cultured on the anoxic environment with 1% O_2_ in DMEM complete medium. After incubation at a fixed time, the cells were fixed with 4% paraformaldehyde and stained with 0.1% ARS (ZY121105, Zeye Biotechnology Co., Ltd., Shanghai) and an ALP detection kit (Beijing Baiolaibo Technology Co., Ltd., Beijing) after 14 days of culture. In addition, the quantative data of Ca nodulus was analyzed by imageJ software.

### Western Blot

For protein analysis, fibroblast cells were rinsed in PBS and lysed using the radioimmunoprecipitation assay (RIPA) buffer containing 1% (v/v) phenylmethylsulfonyl fluoride (PMSF, P7626; Sigma-Aldrich). After the protein supernatant was harvested and detected by the bicinchoninic acid (BCA) protein assay kit (ab102536; Abcam, Cambridge, United Kingdom). The protein was denaturated in water bath (at 95°C, for 5 min) after adding a protein loading buffer. The cells lysates were then processed with 12% SDS-PAGE gel electrophoresis at 120 V for 1 h. The polyvinylidene fluoride (PVDF) membrane (Millipore, Billerica, MA) was used to load proteins. Then Western blocking buffer was used to treat the PVDF membranes (at room temperature), which were incubated by anti-LC3B antibody (Abcam, ab51520), anti-P62 antibody (Abcam, ab101266), anti-Beclin1 antibody (Abcam, ab207612), and anti-Ki67 antibody (Abcam, ab243878) primary antibodies overnight at 4°C. The next day, the tris-buffered saline (TBS) containing 0.1% Tween 20 (TBST) buffer was used to rinse the PVDF membranes twice, then he PVDF membranes incubated for 2 h by the secondary antibodies (1:2000; Proteintech, Rosemont, IL) in a Western secondary antibody dilution buffer. The Tannon 5,200 Multi image analysis system (Tanon Technology Co., Shanghai, China) was used to test all blot intensities. The Quantity One software (Bio-Rad, Hercules, CA) was used to test band density, and blots were carried out in triplicate.

### RT-qPCR

Total RNA was extracted using TRIzol reagent (Invitrogen) after 14 days of culture. cDNA was synthesized using the PrimeScript™ RT reagent kit (Takara). Amplification reactions were set up in 96-well plates using iTaq™ SYBR Green Super Mix (BIO-RAD), to which gene-specific forward and reverse PCR primers were added. These analyses were performed to detect Runx2 and osteopontin (OPN) mRNA expression, and *β*-actin was used as an internal control. The primer sequences are listed in Table 1.

**TABLE 1:** Primers for qRT-PCR analysis of gene expression.

Primer	5′ forward 3′	5′ reverse 3′
Runx2	TAC​TGT​CAT​GGC​GGG​TAA​CG	CAC​CTG​CCT​GGC​TCT​TCT​TA
OPN	GAA​CAT​GAA​ATG​CTT​CTT​TCT​CAG	TCC​ATG​AAG​CCA​CAA​ACT​AAA​CTA
*β*-Actin	GCT​TCT​AGG​CGG​ACT​GTT​AC	CCA​TGC​CAA​TGT​TGT​CTC​TT

### PEGDA/TCS Drug Delivery System Established for Evaluating the Synergistic Effect of Res and ANG2

PEGDA/TCS drug delivery system: The 15 g freeze-dried PEGDA/TCS hydrogel was immersed in 20 ml of ANG2, ANG2/Res, and ANG2/Res/3-MA solution for 5 h; the ANG2 concentration was 400 ng/ml; the Res concentration was 800 μM; and the 3-MA concentration was 20 mM. As the hydrogel was freeze-dried, the hydrogel possess vast pore to absorb the solution, when the solution was added into the hydrogel, the solution was almost absorbed into the hydrogel interior. After incubation, the samples were molded into cylinders (φ3×5 mm), and the PEGDA/TCS drug delivery system was collected for Co60 irradiation at 24 kGy. In addition, the release property of PEGDA/TCS hydrogel was tested by putting the 0.1 g PEGDA/TCS hydrogel which contained ANG2/Res into 20 ml PBS solution. At the designed time, 10 ml solution was collected and flesh PBS was replenished. The collected PBS was analyzed by UV/vis (determination wavelength is 305 nm) method for Res and BCA protein method for ANG2.

### *In vivo* Implantation and Surgical Procedure

This study was approved by the Jinan University Laboratory Animal Ethics Committee, Guangzhou, China (20170912214418). A total of 44 male SD rats aged 4–8 weeks were divided into the control, ANG2, ANG2/Res, and ANG2/Res/3-MA groups. In which, the control group means only PEGDA/TCS scaffold. All rats underwent surgery for the creation of a 3 mm-diameter hole in the left tibia under anesthesia using ketamine at 125 mg/kg, and then the scaffolds (diameter, 3 mm; length, 5 mm) were placed into the defect. Then, the scaffolds were wrapped with the surrounding fibrous tissue. Ampicillin was given to every rat at a dose of 0.1/100 g. The sufficient tap water and chow were provided ad libitum. The rats were permitted relative free ambulation, and the rats were sacrificed by cardiac puncture under diethyl ether anesthesia at 8 weeks after surgery. Specimens were fixed in 10% formalin. Histology, immunofluorescence, immunohistochemistry, and Masson staining were performed to evaluate new bone and vessel formation.

### Histology, Immunofluorescence, Immunohistochemistry, and Masson Staining

At the end of the 8th week after implantation, 11 SD rats in each group were sacrificed by cardiac puncture under diethyl ether anesthesia, and the tibia was then dissected. After gross observation, each rat tibia with an implant was fixed in buffered formalin (10%) for 24 h and then decalcified in 15% EDTA for one month. The EDTA solution was replaced every two days. After decalcification, the tibia was halved transversely by a scalpel, dehydrated in ethanol solutions, embedded in paraffin, and sectioned.

Conventional hematoxylin and eosin (H&E) staining was conducted complied with the normal operation. The sections were stained with H&E as well as Masson’s trichrome and observed by fluorescence microscopy (BX51, Olympus).

For immunohistochemistry staining, the paraffin section was immersed in xylene solution twice, 15 min each time. Then use 100, 95, 90, 80, and 70% gradient alcohol was hydrated, each gradient was hydrated for 10 min, and then washed with PBS for three times, 10 min each. The sections were soaked in 3% H_2_O_2_ solution for 10 min to remove endogenous catalase and digested with citric acid solution for 15 s in boiling temperature to expose the site of the antigen. Then, the sections were blocked with goat serum for 15 min at room temperature. Monoclonal antibody against OCN (Thermo Fisher, AB_2064891) and CD31 (Thermo Fisher, AB_2549792) were cultured with section and stored overnight in a refrigerator at 4°C. The specimens were subsequently incubated with secondary antibody against mouse IgG (1:500) for 30 min at 37°C, followed by incubation with streptavidin-HRP and diaminobenzidine (DAB) substrate and counterstaining with hematoxylin solution.

For immunofluorescence staining, the sections were non-specific blocked for 40 min by 3% bovine serum protein. Then the sections were blocked by incubation with mouse primary antibody against CD31 (Thermo Fisher, AB_928130). After incubation, the sections were rinsed three times with PBS for 10 min each. Then, rhodamine-labeled goat secondary antibody (Thermo Fisher, AB_228357) was added and incubated for 30 min, followed by washing three times with PBS for CLSM (Confocal laser scanning microscopy, LSM 880, Zeiss) observation.

### Statistical Analysis

All the results are displayed as the mean ± standard deviation for at least three independent experiments. Student’s t test was used to evaluate comparisons, and GraphPad Prism software 7.0 (GraphPad, San Diego, CA) was used for one-way analysis of variance. Differences were considered significant at *p < 0.05 and **p < 0.01.

## Results and Discussion

### BMSC Identification

**FIGURE 1:**
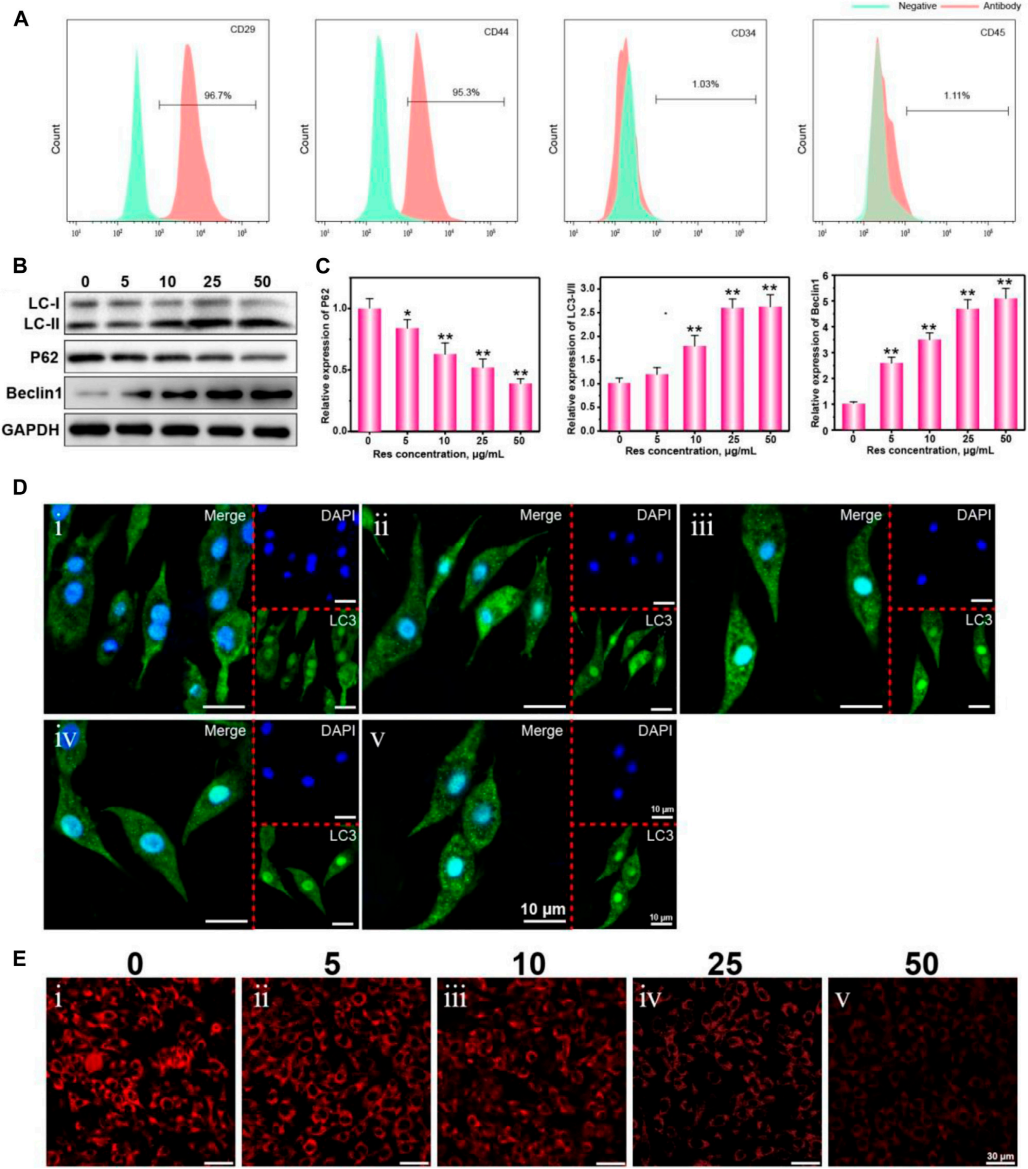
The growth morphology **(A)**, cell viability **(B)**, real-time cell growth curve **(C)**, WB of Ki67 **(D)**, and statistical analysis **(E)** of BMSCs treated with Res or 3-MA. The values are represented as the mean ± SD (*n* = 3). *p < 0.05, **p < 0.01 vs control (without Res and 3-MA).

**FIGURE 2:**
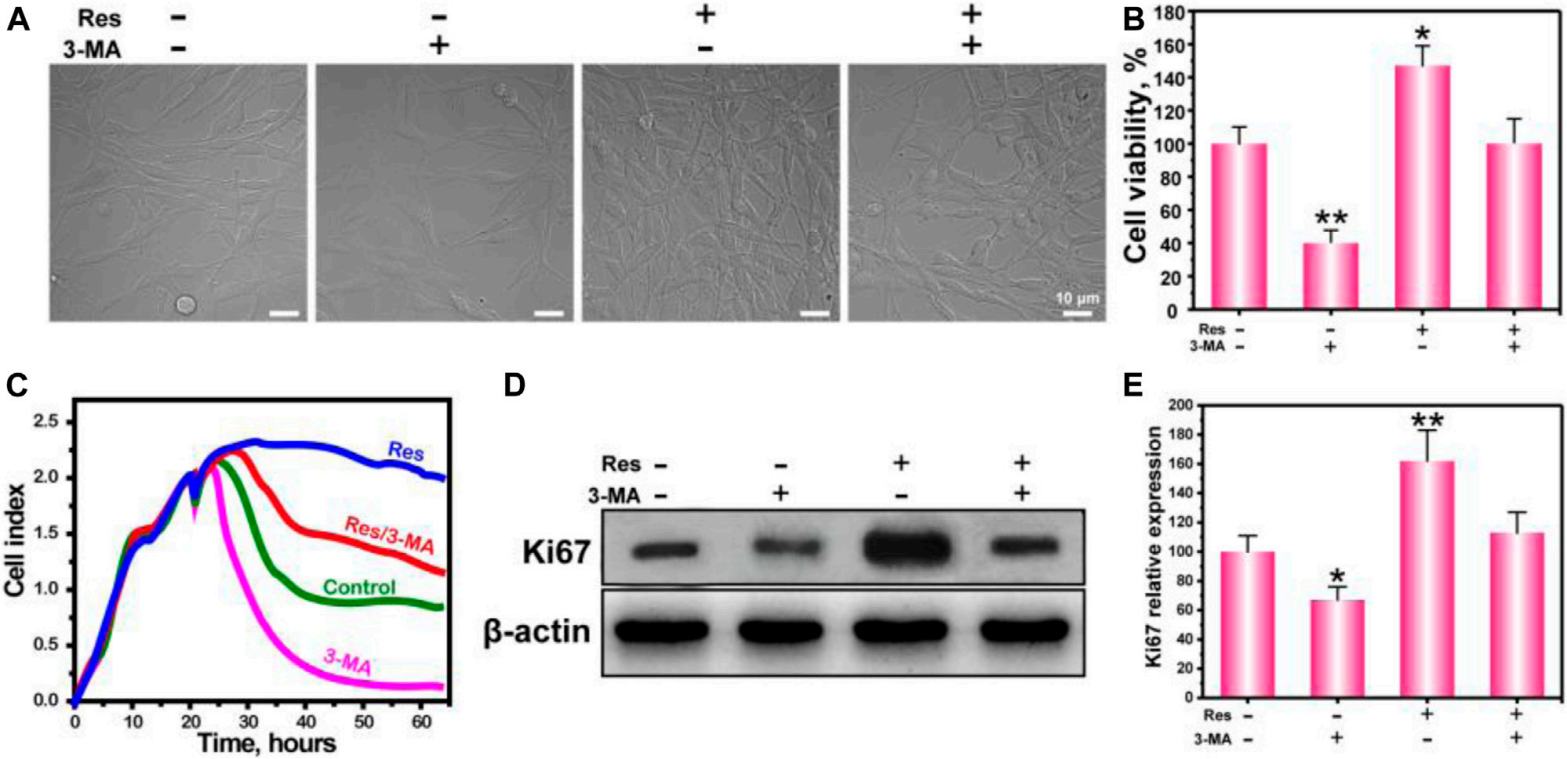
The biomarkers CD29, CD44, CD34, and CD45 were identified by flow cytometry **(A)**. Western blot analysis the expression of autophagy characteristic proteins LC-Ⅰ, LC-Ⅱ, P62, and Beclin1 **(B),** and statistical analysis **(C)**, CLSM observation of the LC3 puncta **(D),** and ROS fluorescence **(E)** in BMSCs treated with different concentrations of Res (0, 5, 10, 25, 50 μg/ml). The values are represented as the mean ± SD (*n* = 3). *p < 0.05 and **p < 0.01 vs control group (Res concentration is 0 μg/ml).

**FIGURE 3:**
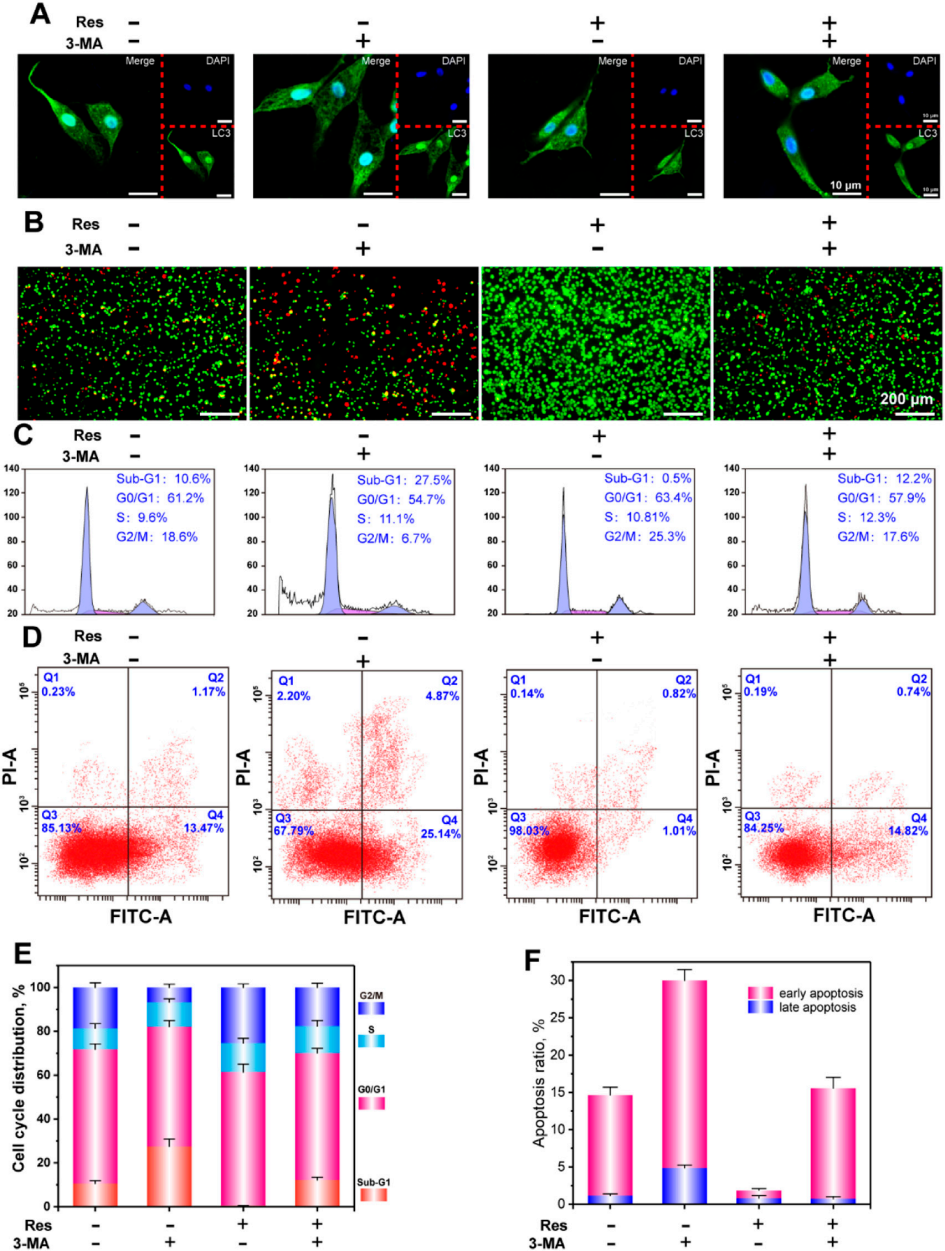
CLSM observation of LC3 puncta **(A)**, AO-EB staining **(B)**, flow cytometry analysis of the cell cycle **(C)**, apoptosis **(D)**, and statistical analysis of the cell cycle **(E)**, and apoptosis **(F)**. The values are represented as the mean ± SD (*n* = 3). *p < 0.05, **p < 0.01 vs. control (without Res and 3-MA).

BMSCs derived from the bone marrow of rats were isolated, cultured, and identified. Surface marker proteins of BMSCs were identified by flow cytometry. Mononuclear cells in the bone marrow of rats were isolated by the Percoll density-gradient centrifugation method and induced *in vitro* to obtain rat bone marrow-derived BMSCs. The isolated BMSCs show adherent growth in the routine culture environment (Figure 1A). The isolated BMSCs in this experiment show high CD29 and CD44 expression and low CD34 and CD45 expression (Figure 2A). They can be differentiated into osteoblasts under induction *in vitro* (indicated by calcium nodule formation) (Figure 3B). The isolated BMSCs met the minimal standards of pluripotent mesenchymal stem cells proposed by the International Society for Cellular Therapy and can continuously be used in subsequent experiments (Saeed et al., 2012).

### Regulation of BMSC Autophagy and ROS Levels in a Hypoxic Environment by Res

To confirm the effect of Res on the autophagy ability of BMSCs in a hypoxic environment, BMSCs were cultured in a hypoxic and serum-free environment for 6 h. Autophagy proteins, including LC3-I, LC3-II, p62, and Beclin1, were detected by WB. The increase in the LC3-II/LC3-I (LC3-II/I) ratio indicates an increase in the autophagy level, and p62 is a marker of autophagic degradation (Mizushima and Yoshimori, 2007). Figure 2B shows that as the Res concentration increased, the protein expression level of p62 gradually decreased, while the LC3-II/I ratio and the relative expression level of Beclin1 gradually increased. These results indicate that the autophagy ability of BMSCs after Res treatment significantly increased in a dose-dependent manner (Gurusamy et al., 2010; Morselli et al., 2010). Figure 2C shows that the grayscale density levels of the bands for LC3-II/I, Beclin1, and p62 changed as the Res concentration increased. To more intuitively observe the increase in BMSC autophagy by Res in a hypoxic environment, BMSCs were transfected with a GFP-LC3 plasmid. Because LC3 forms aggregates during autophagy, the aggregation of fluorescence-labeled LC3 proteins reflects the intensity of autophagy in the cells. Figure 2D shows the presence of a small number of autophagy puncta in the cytoplasm in the hypoxic culture environment, indicating that hypoxia could cause BMSCs to undergo autophagy to a small degree. After the addition of Res, the number of autophagy puncta in BMSCs significantly increased in a dose-dependent manner. When the Res concentration increased from 5 to 50 µg/ml, the number of autophagy puncta in BMSCs gradually increased.

Next, we investigated the effect of Res on the ROS level in BMSCs cultured under hypoxia. Figure 2E shows the relationship between the intracellular ROS levels in BMSCs in the hypoxic environment and the ROS concentrations. The results indicate that BMSCs produced a large amount of intracellular ROS in the hypoxic environment, severely damaging the normal physiological functions of the cells. As the Res concentration increased, the intracellular ROS levels in BMSCs gradually decreased. It is possible that since ROS are mainly derived from the mitochondrial electron transport chain and enzymatic reactions, as autophagy increases, damaged mitochondria are engulfed to minimize ROS production (Rouschop et al., 2009; Rouschop and Wouters, 2009).

### Regulation of BMSC Proliferation by Res Through the Autophagy Pathway in a Hypoxic Environment

To investigate whether Res can regulate proliferation by affecting BMSC autophagy in a hypoxic environment, we observed cell growth under a light microscope (Figure 1A), measured cells proliferation by 3-(4,5-dimethylthiazol-2-yl)-2,5-diphenyltetrazolium bromide (MTT) assay (Figure 1B), tested real-time cell growth (Figure 1C) and verified the proliferation marker Ki67 expression (Figure 1D). The results show that BMSCs appeared spindle-like or polygonal in shape and adherent growth. The cell density in the Res group is significantly higher than that in the other three groups. These results indicate that Res promoted BMSC proliferation in a hypoxic environment.

To further quantify the regulation of BMSC proliferation by Res, BMSCs were cultured in a hypoxic environment for 24 h, and cell proliferation was measured by MTT assay. In addition, 3-MA was added into the experiment groups, in which 3-MA was served as the specific inhibitor of autophagy to verify the autophagy effect of Res. The results show that cell proliferation in the 3-MA group is significantly less than that in the control group, and cell proliferation in the Res group is significantly greater than that in the control group. In addition, cell proliferation after the addition of Res and 3-MA decreased to a level similar to that in the control group. The MTT assay indicates that Res improved BMSC proliferation in a hypoxic environment by up-regulating autophagy.

To more intuitively show the growth condition of BMSCs in a hypoxic environment and the improvement in BMSC proliferation by Res through the autophagy pathway, the growth curve of BMSCs in a hypoxic environment was drawn. After the cells were cultured for 20 h, the culture medium was replaced with anaerobic medium, and the cells were continuously cultured in a hypoxic environment. The results indicate that BMSC growth was significantly suppressed, and the growth curve dropped. After being cultured with Res, BMSC growth was no longer restricted by the hypoxic environment, and the growth curve became a nonstandard S-shaped curve. In the culture environment with Res and 3-MA added together, BMSC growth was suppressed again, indicating that the improvement in BMSC growth and proliferation by Res depended on the autophagy pathway. To provide more persuasive evidence for this proliferation phenomenon, the expression level of Ki67 was detected by WB (Figure 1D). Ki67 is a cell proliferation-associated nuclear antigen. Its function is closely associated with mitosis and is indispensable in cell proliferation (van Oijen et al., 1998). The results of WB experiments show that BMSCs in the Res group expressed higher levels of Ki67. After the addition of Res and 3-MA together, the Ki67 expression level in BMSCs was significantly suppressed to a level similar to that in the control group. These results are consistent with the results of our MTT assay and further confirm that Res regulated BMSC growth and proliferation in a hypoxic environment through the autophagy pathway.

### Regulation of Apoptosis and Cell Cycle of BMSCs by Res in a Hypoxic Environment Through the Autophagy Pathway

**FIGURE 4:**
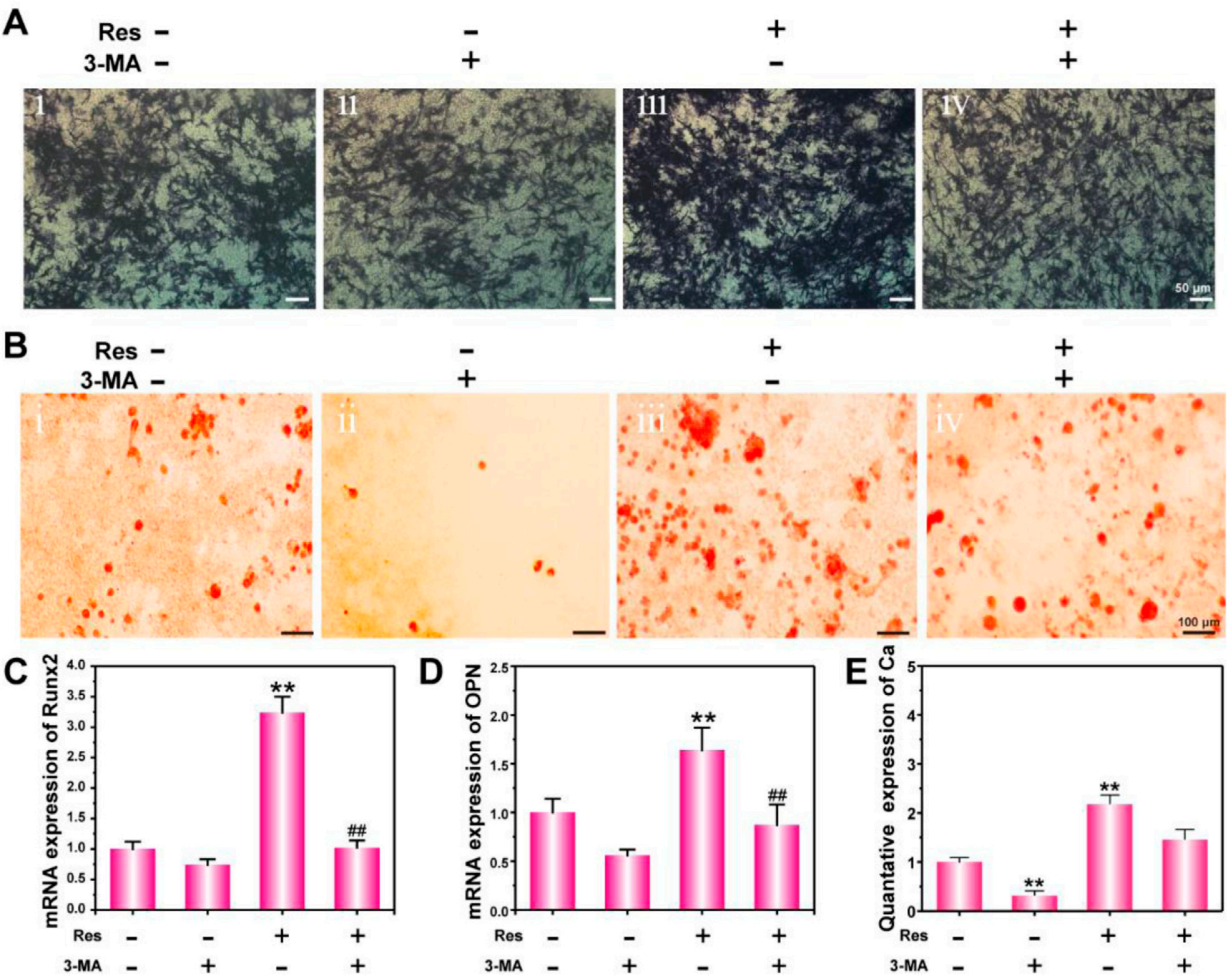
Osteogenic differentiation of BMSCs treated with Res and 3-MA for 14 days. ALP activity detection **(A)** and ARS staining **(B)**. mRNA expression of Runx2 **(C)** and OPN **(D)** detected by q-PCR. The quantative data of Ca nodulus analyzed by ARS staining using ImageJ software **(E)**. The values are represented as the mean ± SD (*n* = 3). *p < 0.05, **p < 0.01 vs. control (without Res and 3-MA); ^#^ *p* < 0.05, ^##^ *p* < 0.01 vs. Res group.

To further investigate the intrinsic mechanism underlying the regulation of BMSC survival by Res in the hypoxic environment, the apoptosis and cell cycle of BMSCs were detected with flow cytometry. Figure 4A shows the regulation of BMSC autophagy by Res in the hypoxic environment and the autophagy-inhibiting effect of the autophagy inhibitor. In the hypoxic environment, a small degree of autophagy was induced in BMSCs, and this degree of autophagy apparently was not sufficient to allow cells to survive. After the addition of 3-MA or Res separately, the number of autophagic vesicles decreased or increased, respectively. After the addition of both 3-MA and Res, the number of autophagic vesicles in BMSCs decreased. These results indicate that 3-MA and Res had inhibitory and activating effects on autophagy, respectively.

Figure 4B shows the AO/EB live/dead staining results of BMSCs in the hypoxic environment. AO can penetrate cells with intact cell membranes and intercalate into nuclear DNA to emit bright green fluorescence. EB can only penetrate cells with damaged cell membranes and intercalate into nuclear DNA molecules to emit orange fluorescence. In hypoxia, BMSCs exhibited overall green staining with localized orange staining in cells, indicating that a hypoxic environment caused a certain level of damage in BMSCs. After 3-MA treatment, BMSC damage was further aggravated, and the morphology of some cells was round or shrunken with a lumpy structure, indicating that some cells had entered the late stage of apoptosis or died. The main reason for this result was that BMSC autophagy was suppressed, so cells could not defend themselves against the hypoxic environment and underwent apoptosis. In the Res treatment group, BMSCs showed overall green staining, indicating that apoptosis induced by hypoxia was suppressed. The application of Res and 3-MA together caused BMSCs to exhibit obvious apoptosis. These results indicate that Res can inhibit BMSC apoptosis in a hypoxic environment through the autophagy pathway.

Figure 4C shows the cell cycle results of BMSCs in the hypoxic environment. Figure 4E shows the statistical analyses of the percentage of cells in each phase of the cell cycle. The sub-G1 phase indicates small fragments of damaged DNA and breakage produced after apoptosis. In the hypoxic environment, the percentage of BMSCs in the sub-G1 phase was 10.6%, whereas the percentage of BMSCs in the sub-G1 phase in the 3-MA treatment group was 27.5%. These results indicate that inhibition of BMSC autophagy in a hypoxic environment could promote apoptosis. The percentage of cells in the sub-G1 phase in the Res and Res/3-MA treatment groups was 0.5 and 12.2%, respectively. These results indicate that Res up-regulated autophagy in BMSCs in the hypoxic environment to inhibit cell apoptosis.

The S and G2/M phases are the two most important stages in cell proliferation (Paterlini et al., 1993). These two stages are the periods when cells are undergoing complex and active molecular-level changes. The experimental results show that the percentage of cells in the S and G2/M phases was significantly higher in the Res group than in the other three groups. These results are consistent with the results of our above proliferation experiments and further confirm the phenomenon that Res increased BMSC proliferation in the hypoxic environment through the autophagy pathway.

To more precisely understand how Res helped BMSCs avoid apoptosis, cell apoptosis was detected using flow cytometry (Figures 4D,F). The results show that in a hypoxic environment, early apoptosis plus late apoptosis accounted for 14.64% of the BMSCs. After Res treatment, this proportion decreased to 1.83%. After Res/3-MA treatment, this proportion increased to 15.56%. These results indicate that Res enhanced BMSC autophagy to decrease early and late apoptosis in BMSCs.

### Regulation of BMSC Osteogenesis Differentiation in a Hypoxic Environment by Res Through the Autophagy Pathway

The effect of Res on the osteogenic differentiation of BMSCs under hypoxia was further investigated *in vitro*. To analyze their protein and calcification levels, BMSCs were subjected to ALP staining and ARS staining. At the gene expression level, the osteogenic differentiation early stage indicator Runx2 and the middle-stage indicator OPN were detected using q-PCR. Figure 3A shows the ALP staining results of BMSCs in a hypoxic environment. The results show that the ALP expression level in the Res treatment group was the highest, whereas the ALP expression level in the Res/3-MA treatment group was significantly decreased. Figure 3B shows that in the Res treatment group, here, dark crystalline clumps with different sizes were observed under the microscope, which were calcified nodules formed in the early stage. The size and area of these clumps were significantly greater in the Res group than in the other three groups. In addition, the quantitative data of Ca nodulus was shown in Figure 3E. Because Runx2 is a very early marker of the osteogenic differentiation of BMSCs and its up-regulation usually only lasts for approximately 3 days, the expression level of the Runx2 gene in BMSCs was detected on day 3 (Figure 3C). The results show that on day 3, the Runx2 expression level in the Res group was significantly higher than that in the other three groups. The OPN middle-stage marker of osteogenic differentiation of BMSCs was detected on day 7, showing a significantly higher OPN level in the Res group than in the other three groups. These results indicate that Res increased the osteogenic differentiation of BMSCs in the hypoxic environment. In the Res/3-MA treatment group, the osteogenic differentiation markers in BMSCs were significantly lower than those in the Res treatment group. These results indicate that in a hypoxic environment, Res can increase the osteogenic differentiation of BMSCs by increasing cell autophagy. In addition, the inhibition of autophagy can inhibit the osteogenic differentiation of BMSCs.

### Regulation of HUVEC Survival in a Hypoxic Environment by Res and ANG2 Through the Autophagy Pathway

ANG2 is a very important cytokine in the early stage of angiogenesis. ANG2 can activate HUVECs into an unstable state to achieve endothelial cell permeability and capillary remodeling. However, the function of ANG2 in endothelial cells in a hypoxic environment is a double-edged sword: while it increases the activation state of endothelial cells to facilitate endothelial cell remodeling (Veikkola and Alitalo, 2002), it also reduces endothelial cell homeostasis, especially in a hypoxic environment (Maisonpierre et al., 1997). Therefore, the issue of endothelial cell stability and survival becomes particularly important.

**FIGURE 5:**
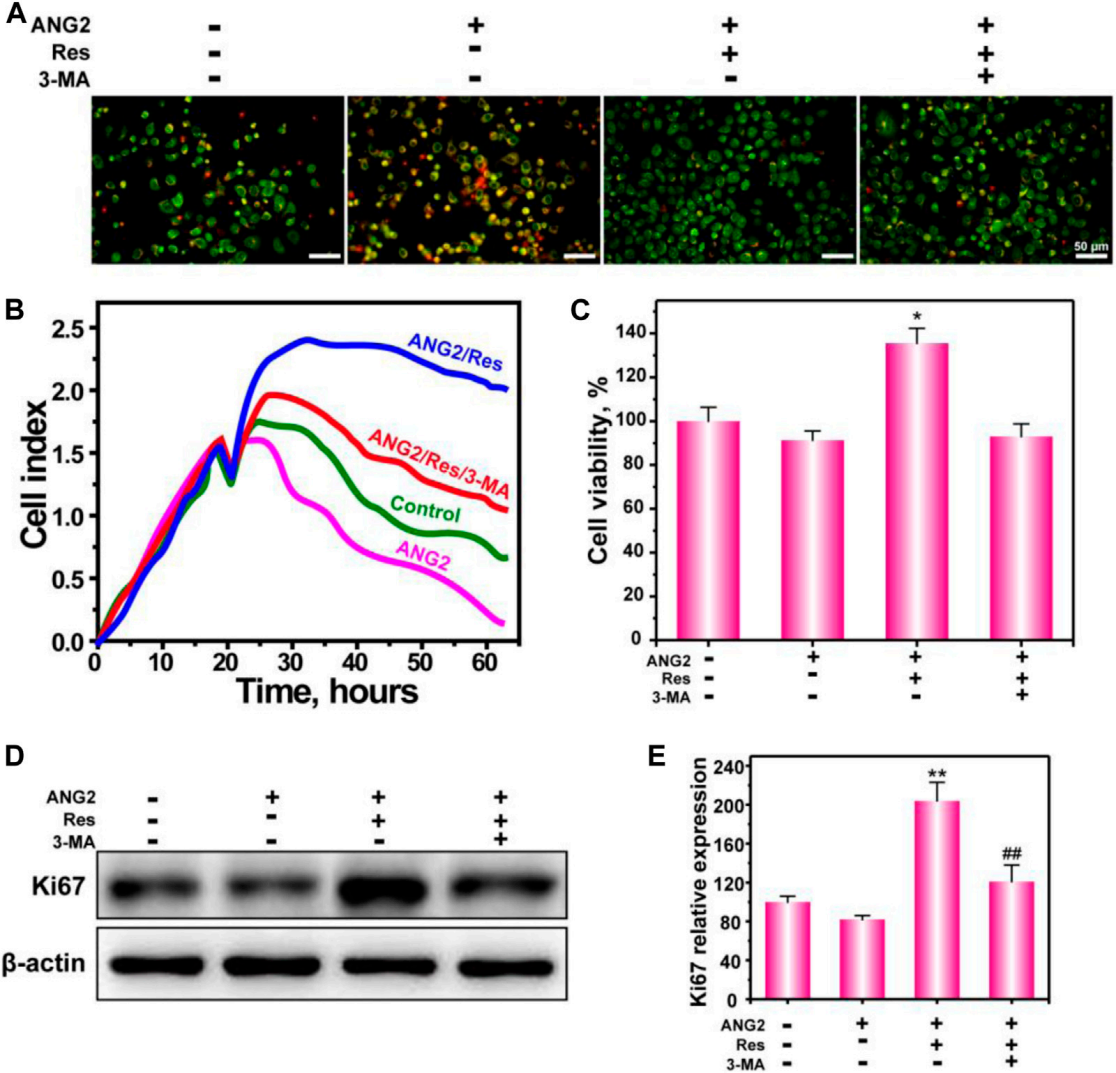
Survival of HUVECs treated with ANG2, Res, and 3-MA in an oxygen-deficient environment. AO-EB staining at 24 h **(A)**, real-time cell growth curve **(B)**, cell viability by MTT assay at 24 h **(C)**, and protein expression of Ki67 **(D)**. The values are represented as the mean ± SD (*n* = 3). *p < 0.05, **p < 0.01 vs ANG2 group; ^#^ *p* < 0.05, ^##^ *p* < 0.01 vs ANG2/Res group.

In this study, we investigated the effect of the combined use of ANG2 and Res on the survival of HUVECs. Figure 5A shows the live/dead staining results of HUVECs. In the hypoxic environment, HUVECs exhibited certain levels of early and late apoptosis and were round or shrunken in shape. After treatment with ANG2, the HUVECs showed greater apoptosis in the hypoxic environment. It is possible that ANG2 itself caused the endothelial cell instability; in the adverse hypoxic environment, endothelial cell apoptosis increased. Fortunately, after treatment with ANG2/Res, the HUVECs exhibited bright green fluorescence, indicating that the combined use of ANG2 and Res could reduce the HUVEC apoptosis caused by hypoxia. When ANG2, Res, and 3-MA were applied together, AO/EB staining of HUVECs revealed orange apoptotic cells. These results indicate that Res decreased the apoptosis caused by ANG2 and hypoxia by increasing autophagy in the endothelial cells. Figure 5C shows the proliferation of HUVECs in a hypoxic environment detected by MTT assay. ANG2 could weakly inhibit HUVEC proliferation, whereas Res/ANG2 treatment increased HUVEC proliferation. HUVEC proliferation decreased again in the Res/ANG2/3-MA treatment group, indicating that Res increased autophagy in HUVECs to maintain HUVEC proliferation in a hypoxic environment.

To more intuitively reflect the growth and proliferation condition of HUVECs in the hypoxic environment, the effect of Res on HUVECs was detected using the real-rime cell growth curve (Figure 5B). The results indicate that the growth of endothelial cells was down-regulated after the addition of ANG2, whereas Res/ANG2 treatment significantly improved endothelial cell proliferation. To more precisely show the effect of Res on HUVEC proliferation in a hypoxic environment at the molecular level, the proliferation marker Ki67 was detected by WB (Figures 5D,E). The results indicate that Res/ANG2 treatment increased the expression level of Ki67 in HUVECs in the hypoxic environment. In the Res/ANG2/3-MA treatment group, the Ki67 expression level was lower than that in the Res/ANG2 group. These results indicate that Res maintained HUVEC growth and proliferation in the hypoxic and ANG2 double-adverse environment through the autophagy pathway.

### Preparation and Characterization of PEGDA/TCS Hydrogel

**FIGURE 6:**
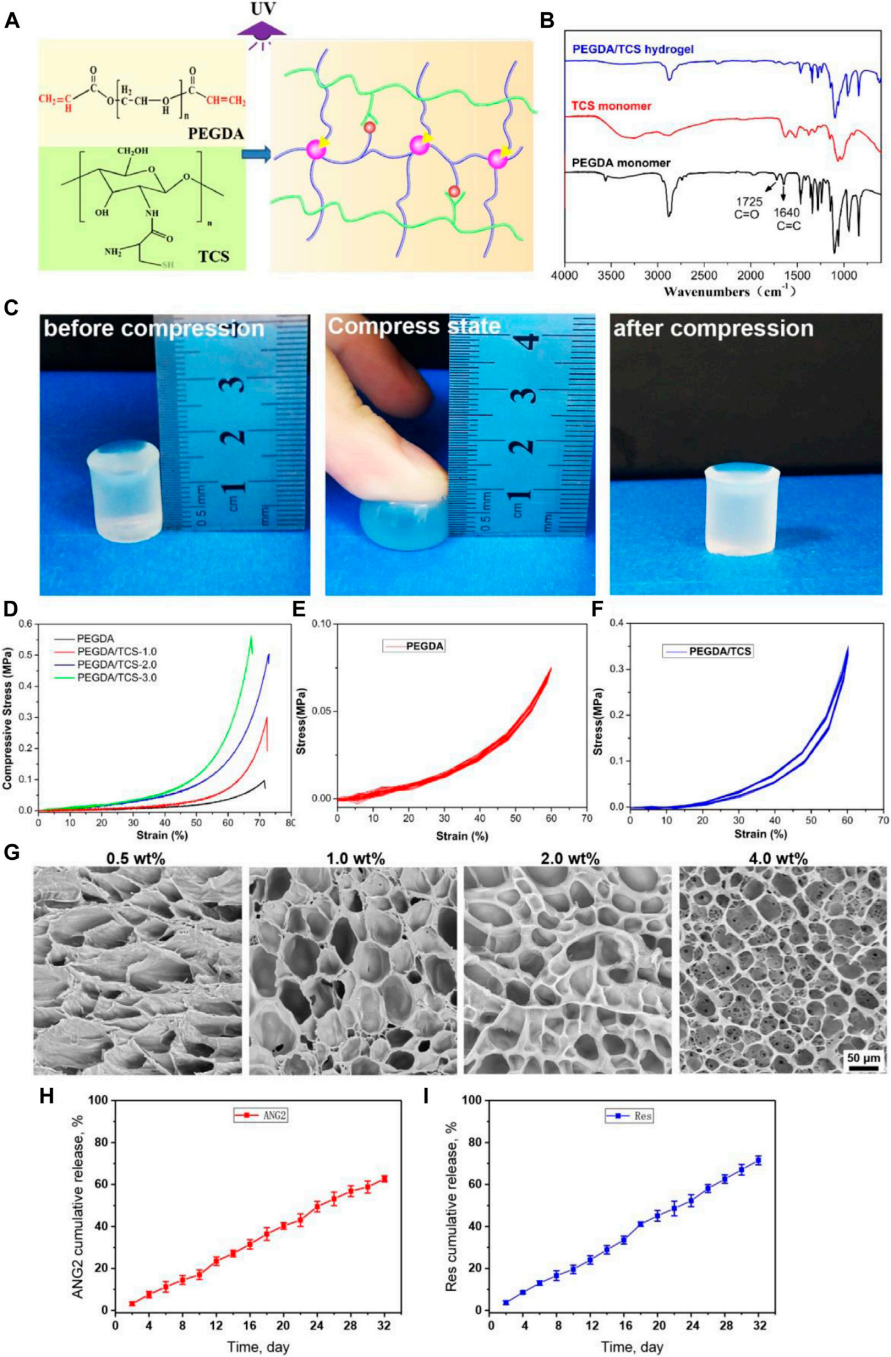
Scheme for the fabrication of PEGDA/TCS hydrogel **(A)**, FTIR spectra **(B)**, schematic of hydrogel compression: **(A)** before compression, (b) under compression and (c) after compression **(C)**, compressive stress-strain curves **(D)**, cyclic compressive stress-strain curves of PEGDA **(E)** and PEGDA/TCS **(F)**, and SEM images of PEGDA/TCS hydrogel for different proportions of TCS **(G)**. The releases curve of ANG2 **(H),** and Res **(I)** from PEGDA/TCS hydrogel. The values are represented as mean ± SD (*n* = 3). The scale bar is 50 μm.

To study the reparative effect of Res and ANG2 combined with tissue engineering scaffold materials in a large-segment bone defect model in a hypoxic environment, PEGDA and TCS were used to prepare a double-network cross-linked hydrogel with a controllable aperture size. In previous research, PEGDA-based hydrogels with highly hydrophilic cross-linked polymers have been applied in regenerative medicine for their highly biocompatible, high porosity, well swollen network structure and proper pore size (Zhang et al., 2016; Tikhonov et al., 2020; Soriente et al., 2021). Here, a schematic diagram of the hydrogel preparation reaction is shown in Figure 6A. The carbon–carbon double bond of PEGDA and the thiol group of TCS were connected by a C–S bond and cross-linked to form the double-network hydrogel.

Figure 6B shows the FTIR characteristics of the PEGDA and TCS monomers and PEGDA/TCS. The C=O peak at 1725 cm^−1^ and the C=C peak at 1,640 cm^−1^ are characteristic peaks of the PEGDA monomer. When the reaction between PEGDA and TCS ended, the C=C peak at 1,640 cm^−1^ disappeared (Huang et al., 2019). This was because the C=C double bond was completely consumed in the cross-linking reaction, and the PEGDA/TCS hydrogel retained other characteristic peaks of PEGDA and TCS. Figure 6C shows an actual picture of the PEGDA/TCS hydrogel. When stress was applied to the hydrogel, the hydrogel greatly deformed but did not break. When the stress was relieved, the hydrogel returned to its original shape. These results indicate that our hydrogel had an excellent ability to resist compression and fatigue under mechanical stimulation. The mechanical compression and cyclic compression properties of the PEGDA/TCS hydrogel were further tested. The results indicate that as the percentage of TCS increased, the compressive strength of TCS increased accordingly. Figures 6E,F show the cyclic compression data of PEGDA and the PEGDA/TCS hydrogel. The results indicate that after many cycles of compression, the compressive strength of the hydrogel was unchanged, suggesting the ability to provide mechanical support in subsequent implantation experiments. Figure 6G shows the appearance of PEGDA/TCS at different ratios. As the TCS content increased, the microstructure of the hydrogel also changed from patchy porous to micro-porous and the aperture shrank. In addition, to assess the loading release profile for PEGDA/TCS hydrogel, the ANG2/Res was absorbed into hydrogel interior (ANG2 loading efficiency is 533 ng/g and Res loading efficiency is 1,066 μM/g). Then the Res and ANG2 release curve was plotted in Figures 6H,I. It can be seen that the Res and ANG2 could both release from the hydrogel and released ratio could up to ∼71.5 and ∼62.7% at 32 days, respectively. In addition, the release profile of Res and ANG2 has almost zero-order release manner may due to the micro-porous structure of PEGDA/TCS scaffold and the polymer chain irregular movement in water swelling process.

### Treatment of Large Bone Defects in Rats Using PEGDA/TCS Tissue Engineering Scaffolds Combined With Res and ANG2

**FIGURE 7:**
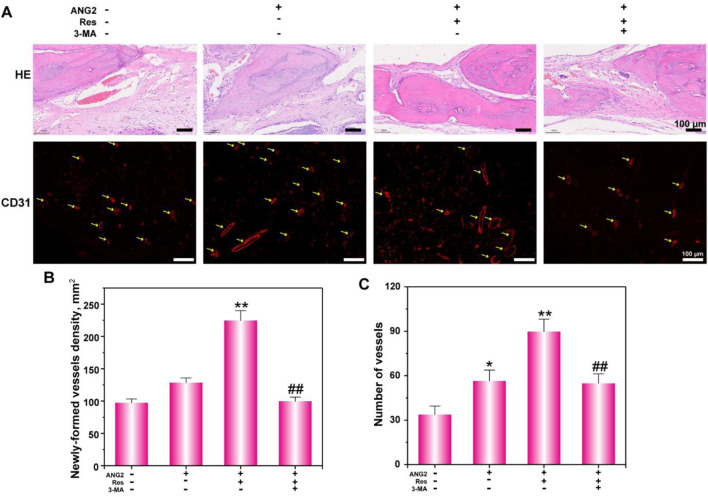
Histological analysis **(A)** of bone defects via H&E staining and CD31 immunofluorescence staining. **(B)** The newly formed vessel density and **(C)** number were determined. The values are represented as the mean ± SD (*n* = 6). *p < 0.05, **p < 0.01 vs. ANG2 group; ^#^ *p* < 0.05, ^##^ *p* < 0.01 vs. ANG2/Res group.

To simulate a relatively hypoxic environment, PEGDA/TCS hydrogels with the smallest apertures were implanted in tibial bone defects in rats. In addition, to evaluate the reparative effect on angiogenesis in the defect, the tissue engineering scaffolds were loaded with both Res and ANG2 before being transplanted into the large tibial defect. At 8 weeks after implantation, the segment of bone containing the defect was collected for H&E staining. Figure 7A shows very favorable bone defect repair in the Res/ANG2/3-MA group. The bone repair rate was much higher in the Res/ANG2/3-MA group than in the other three groups. New bone filled the whole defect area and formed tight connections with old bone, and a very small amount of fibrous tissue filling was observed. In the other three groups (control, ANG2, and Res/ANG2), there was only a small amount of new bone formation, the bone defect area mostly contained fibrous tissue, and there was an obvious lack of wound healing. The newly present fibrous tissues occupied the space of new bone formation and greatly inhibited the rate of bone repair.

To further investigate the state of angiogenesis in the new tissue, CD31 was detected by immunofluorescence. The results show that there was only a small amount of capillary production in the control group, and the total number of capillaries was relatively low. In the ANG2-loaded hydrogel scaffold implantation group, blood vessels with larger lumens were observed, which were mainly attributed to the vascularization effect of ANG2. In the ANG2/Res group, more blood vessels were present, including blood vessels with large lumens, blood vessels with small lumens, and aggregated endothelial cells that were forming capillaries. The number and size of blood vessels in the bone defect area in the 3-MA group were both down-regulated, indicating that Res regulated the angiogenesis of endothelial cells through the autophagy pathway. Figure 7B shows the statistical analysis of the number and density of new blood vessels in the bone defect area. The results show that the combination of Res and ANG2 significantly promoted angiogenesis compared to ANG2 alone. After the addition of 3-MA, both the density and number of blood vessels significantly decreased, indicating that Res assisted ANG2 in promoting angiogenesis through an increase in the local autophagy level in endothelial cells. As for the repair mechanism, only when the cells possessed the basic survival ability, the cells begin play a physiological function, such as BMSCs begin to osteogenesis differentiation, HUVECs begin to form new vessels. Thus, the PEGDA/TCS loaded with Res/ANG2 could accelerate the bone defect repair speed.

**FIGURE 8:**
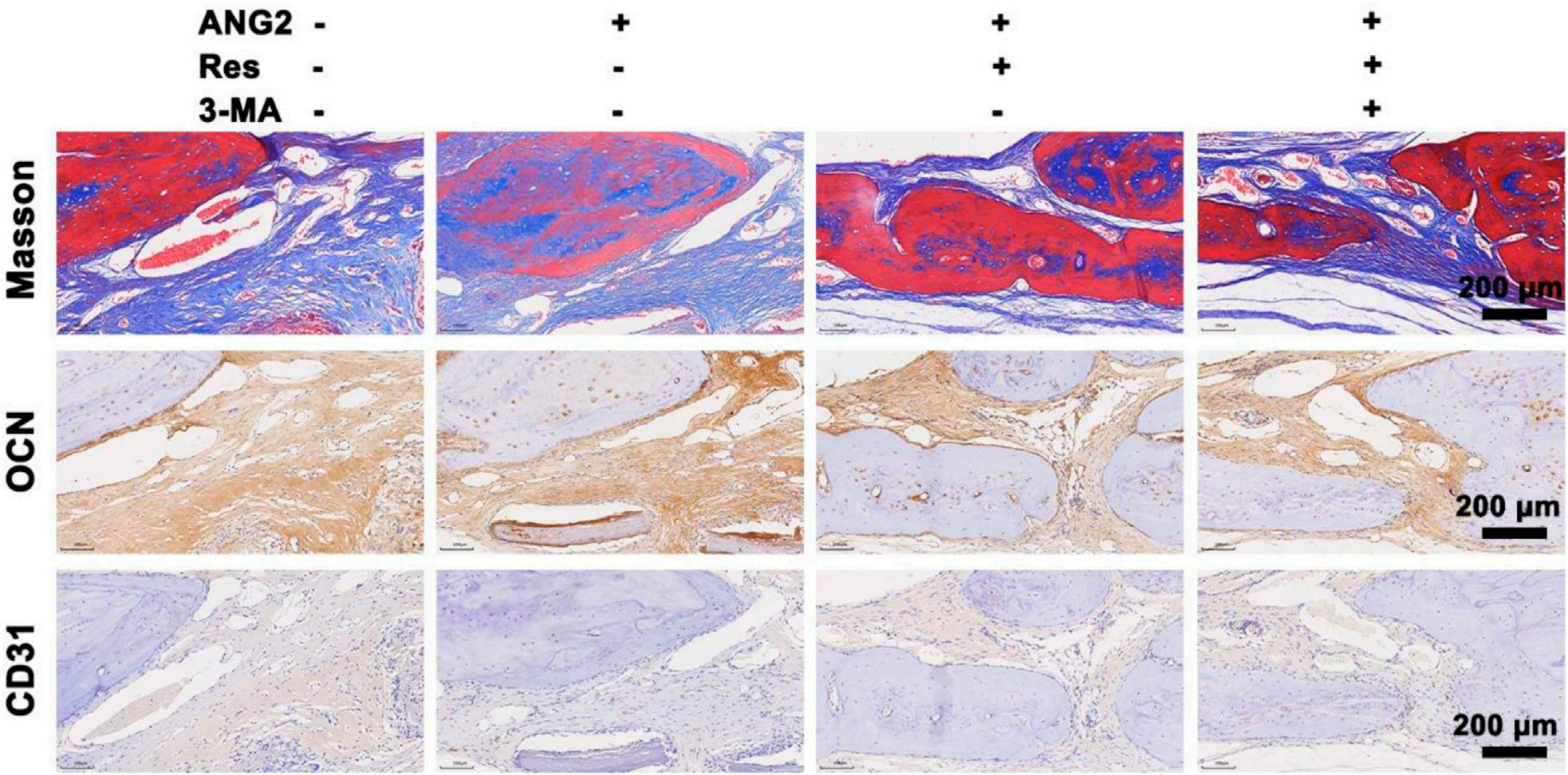
Masson staining and immunohistochemical staining for OCN and CD31 in the bone defect area at the 8th week. The values are represented as the mean ± SD (*n* = 6).

Eight weeks after surgery, the tibial defect location was collected for Masson’s trichrome staining (Figure 8), by which collagen fibers and new bone appear blue, cytoplasm, and fibrous tissue appear red, and cell nuclei appear bluish-black. There was a small amount of new collagen tissue in the control group, and the majority of space was occupied by fibrous tissue and fibrocytes. New collagen in the ANG2 group showed an irregular striped distribution, and there was only a weak connection among them. In the ANG2/Res group, new collagen occupied almost the whole field, with even staining, and the new bone area was significantly larger in this group than in the other groups. In addition, there were bone lacunae, red bone marrow cell accumulation, and blood vessels, indicating the formation of bone marrow cavities and blood vessels in the new bone tissue. In the ANG2/Res/3-MA group, new collagen in the bone defect area was disordered, and there was a small amount of fibrous tissue; in addition, there were no bone lacunae, bone marrow cavities, or blood vessels. The Masson staining results suggest that ANG2 combined with Res achieved new bone formation in the defect area earlier.

To further investigate the ability of Res combined with ANG2 to promote bone formation, the relatively late-stage indicator osteocalcin (OCN) was studied (Boguslawski et al., 2000). The results show that OCN expression in the ANG2/Res group was stronger than that in the other three groups. Fibrous tissues in the ANG2/Res group expressed more OCN. To further study the effect of Res combined with ANG2 on angiogenesis in the bone defect area, CD31 was measured as an indicator of angiogenesis using immunohistochemistry. The results show that there were few capillaries in the control group, the blood vessel lumens were all small, and CD31 expression was very low. In the ANG2 group, the number of blood vessels and the expression of CD31 were increased. This was mainly because ANG2 itself is an angiogenesis factor and ANG2-induced increases in blood vessel size and number are a normal phenomenon. When ANG2 and Res were both added, the number and size of blood vessels in the bone defect area increased, and the highest amount of CD31 was expressed around the vascular lumen. CD31 expression was decreased in the hydrogel group with ANG2/Res/3-MA. The Masson staining and OCN and CD31 immunohistochemical staining results show that Res combined with ANG2 better promoted new bone formation and angiogenesis in the defect area by up-regulating autophagy.

## Conclusion

Hypoxia is a major cause of failure among tissue engineering strategies for treating bone defects. In this work, we prepared PEGDA/TCS hydrogels and adjusted the pore size via the content of TCS. Then, a PEGDA/TCS scaffold for tissue engineering was established. In the *in vitro* experiment, ROS detection, cell viability, flow cytometry and AO-EB staining indicated that Res protected BMSCs and HUVECs against cell damage and apoptosis by activating autophagy in the hypoxic environment. ALP staining, ARS staining and q-PCR indicated that Res could promote BMSC differentiation. In addition, Res maintained HUVEC growth and proliferation in the hypoxic and ANG2 double-adverse environment through the autophagy pathway. In the *in vivo* experiment, Res and ANG2 were cultured in PEGDA/TCS scaffolds and transplanted into the large bone defect area. CD31 immunofluorescence detection suggested that ANG2 combined with Res could induce new blood vessel reconstruction in the defect area earlier through the autophagy pathway. H&E staining showed that new bone tissue in the bone defect area in the ANG2/Res group was almost completely formed and exhibited a network structure at 8 weeks. The results of Masson and immunohistochemical staining suggested that the PEGDA/TCS contained Res and ANG2 tissue-engineered scaffold could promote vascularization and tissue repair in the tibial defect. All these results suggest that Res and ANG2 may be a novel strategy for tissue engineering scaffolds for the targeted therapy of hypoxic bone defects.

## Data Availability

The raw data supporting the conclusions of this article will be made available by the authors, without undue reservation.
