# Correction: Resveratrol and Angiogenin-2 combined with PEGDA/TCS hydrogel for the targeted therapy of hypoxic bone defects via activation of the autophagy pathway

**DOI:** 10.3389/fphar.2025.1597755

**Published:** 2025-09-17

**Authors:** Dehui Fan, Hengping Liu, Zhenning Zhang, Meiyi Su, Zhixian Yuan, Ying Lin, Shuquan Yang, Wenqiang Li, Xintao Zhang

**Affiliations:** ^1^ The Fifth Clinical College of Guangzhou University of Chinese Medicine Guangzhou, Guangdong Second Traditional Chinese Medicine Hospital, Guangzhou, China; ^2^ Beijing University of Chinese Medicine Third Affiliated Hospital, Beijing, China; ^3^ Engineering Technology Research Center for Sports Assistive Devices of Guangdong, Guangzhou Sport University, Guangzhou, China; ^4^ Department of Sports Medicine and Rehabilitation, National and Local Joint Engineering, Research Center of Orthopaedic Biomaterials, Peking University Shenzhen Hospital, Shenzhen, China

**Keywords:** resveratrol, ANG2, autophagy, hypoxia condition, vascularization, bone defect

In the published article, there was an error in Figures 3B, 4A,B as published. Images from different groups were stored in the same folder during image shooting, resulting in the misuse of images**.** The corrected Figures 3B, 4A,B and their captions appear below.

**FIGURE 3:**
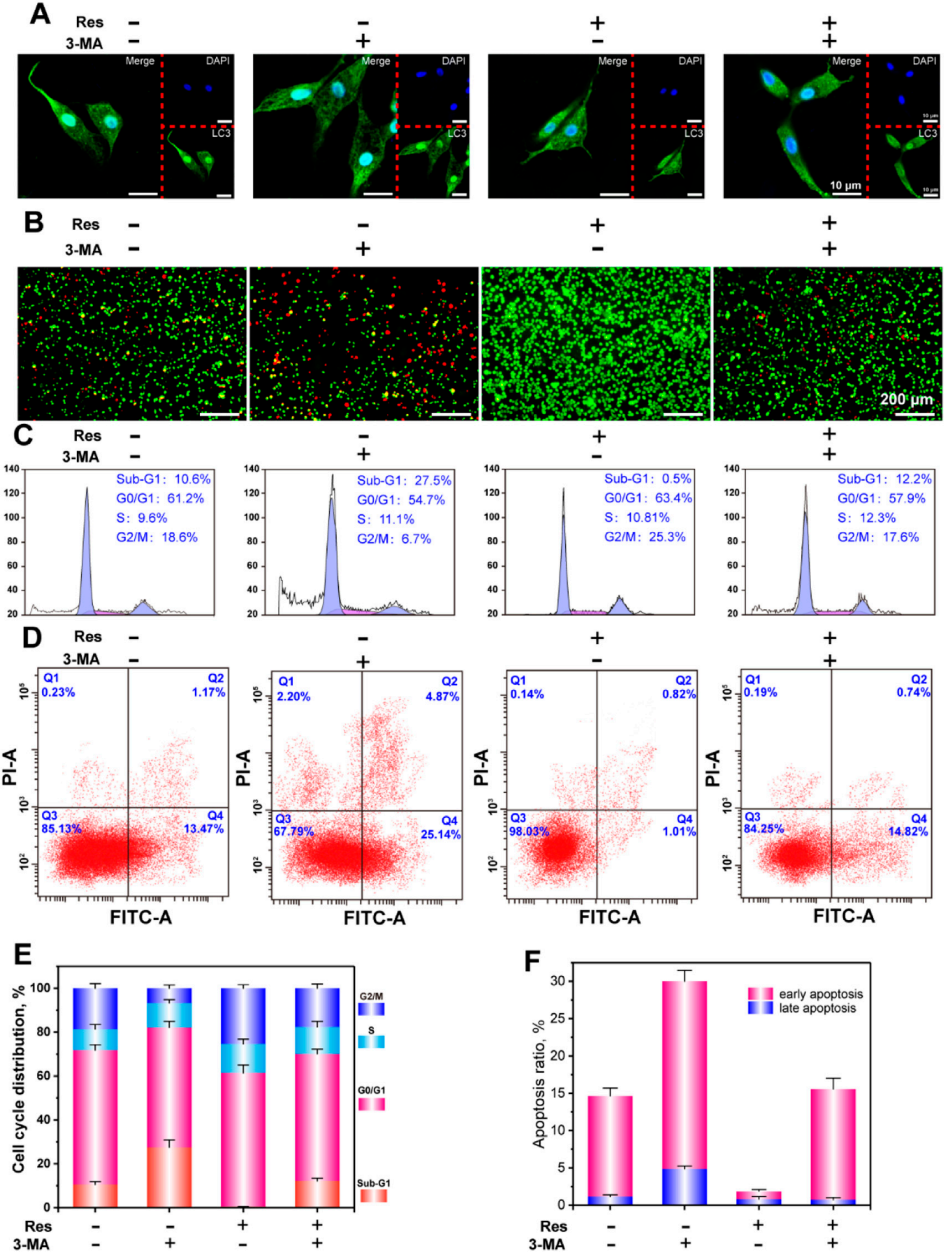
CLSM observation of LC3 puncta **(A)**, AO-EB staining **(B)**, flow cytometry analysis of the cell cycle **(C)**, apoptosis **(D)**, and statistical analysis of the cell cycle **(E)**, and apoptosis **(F)**. The values are represented as the mean ± SD (n = 3). *p < 0.05, **p < 0.01 vs. control (without Res and 3-MA).

**FIGURE 4:**
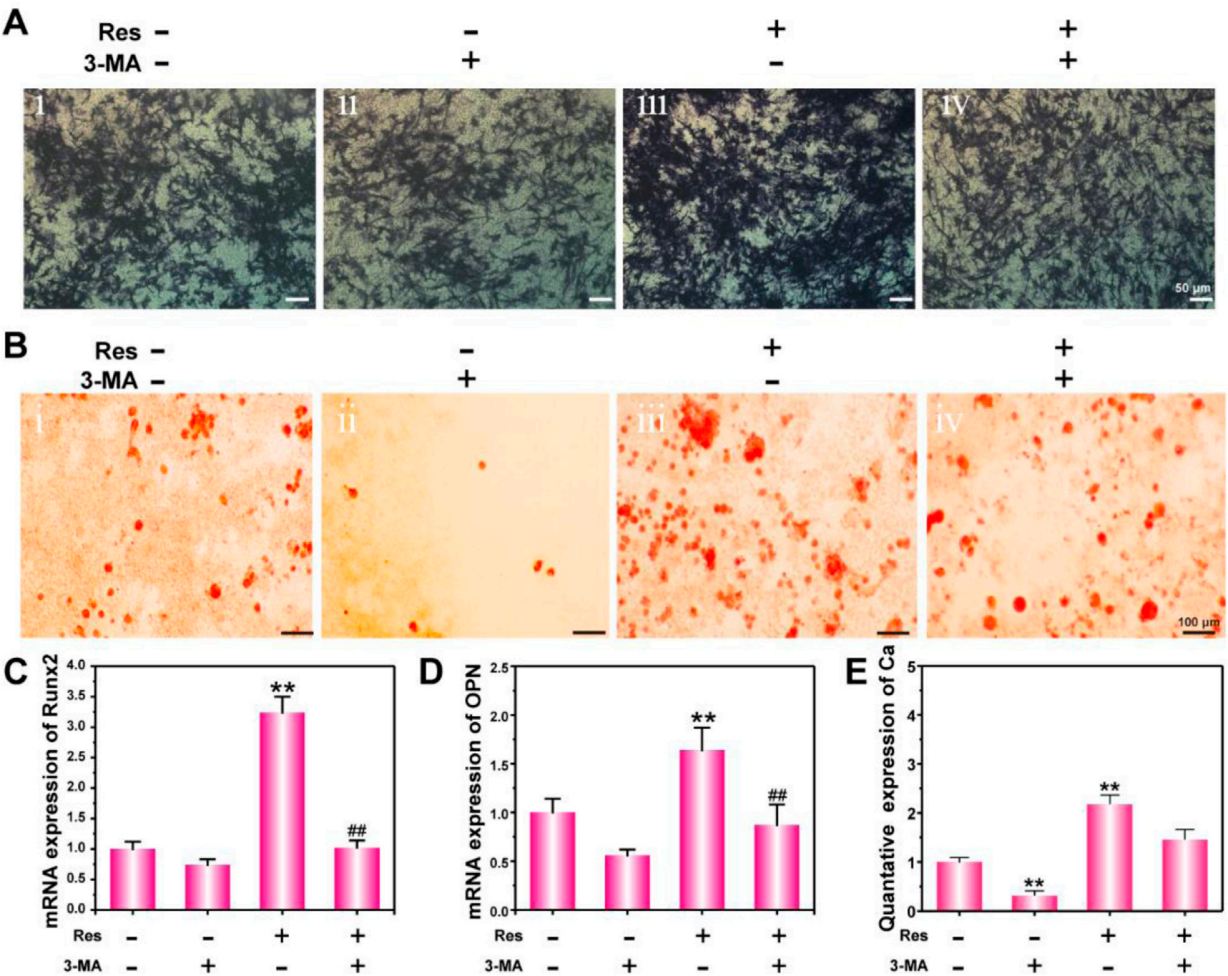
Osteogenic differentiation of BMSCs treated with Res and 3-MA for 14 days. ALP activity detection **(A)** and ARS staining **(B)**. mRNA expression of Runx2 **(C)** and OPN **(D)** detected by q-PCR. The quantative data of Ca nodulus analyzed by ARS staining using ImageJ software **(E)**. The values are represented as the mean ± SD (n = 3). *p < 0.05, **p < 0.01 vs. control (without Res and 3-MA); #p < 0.05, ##p < 0.01 vs. Res group.

In the published article, there was an error in Figures 7A, 8 as published. The H&E, Masson, OCN and CD31 staining images of the animal tissue sections in Figures 7, 8 overlap with those used in our previous published study. This occurred because the animal experiments for both research projects were conducted concurrently, leading to inadvertent misplacement of data files**.** The corrected Figures 7A, 8 and their captions appear below.

**FIGURE 7:**
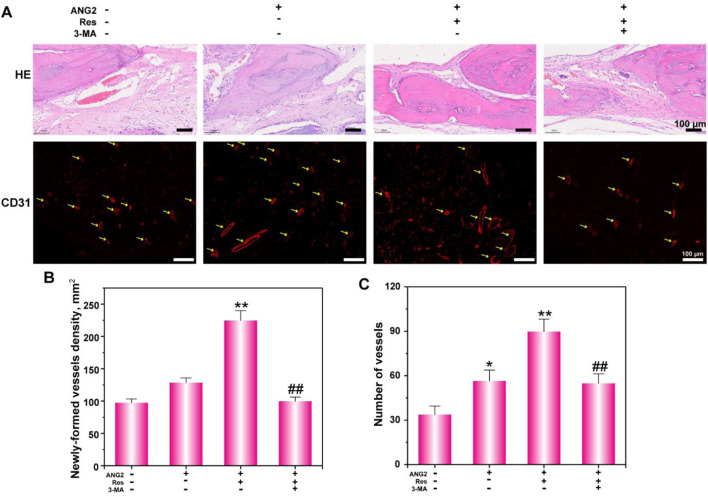
Histological analysis **(A)** of bone defects via H&E staining and CD31 immunofluorescence staining. **(B)** The newly formed vessel density and **(C)** number were determined. The values are represented as the mean ± SD (n = 6).*p < 0.05, **p < 0.01 vs. ANG2 group; #p < 0.05, ##p < 0.01 vs. ANG2/Res group.

**FIGURE 8:**
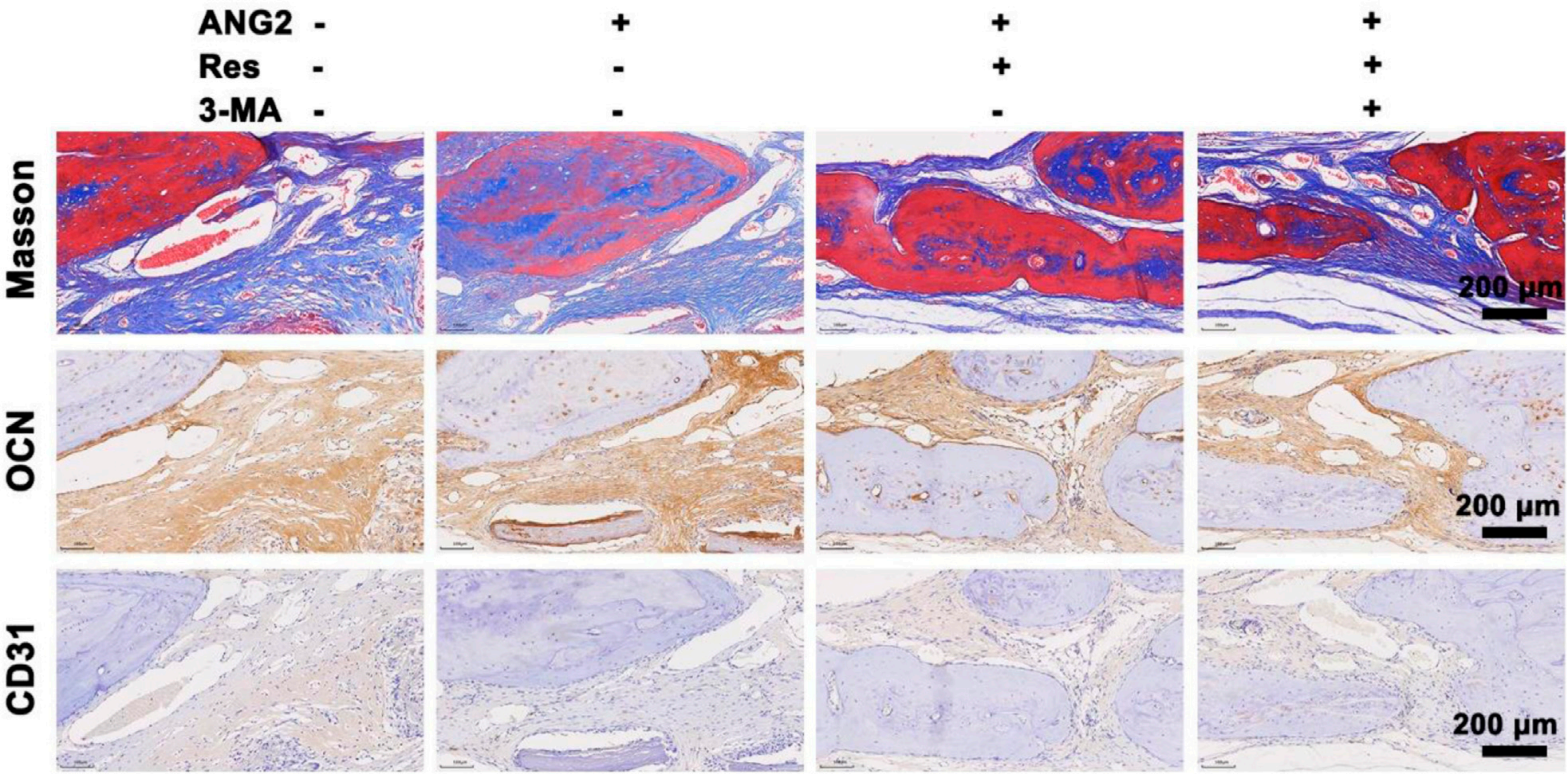
Masson staining and immunohistochemical staining for OCN and CD31 in the bone defect area at the 8th week. The values are represented as the mean ± SD (n = 6).

The original article has been updated.

